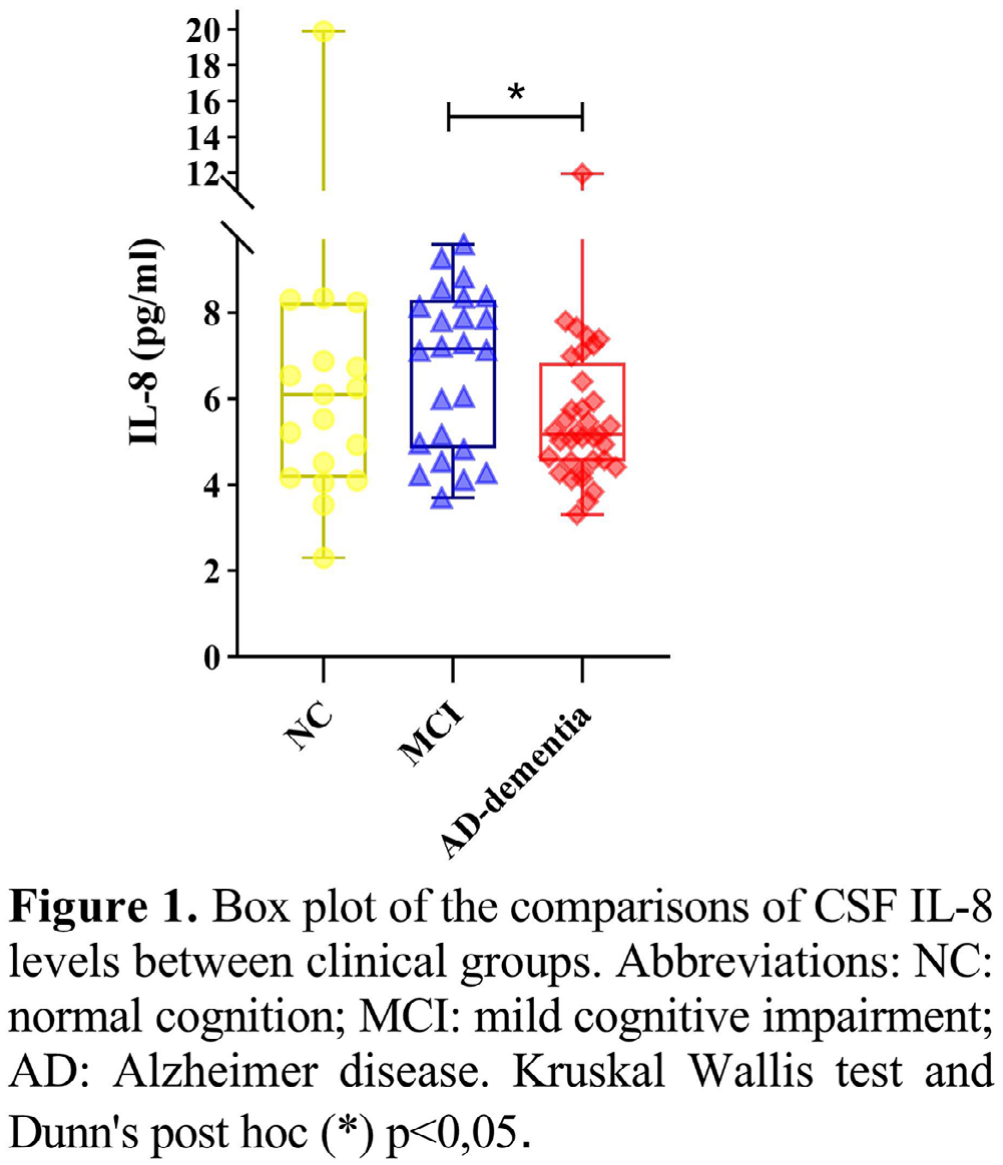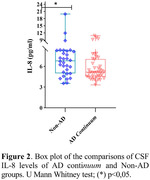# Decreased levels of CSF CXCL8 in clinical and biological profiles of Alzheimer's disease: results of a cohort of Brazilian older adults

**DOI:** 10.1002/alz.092843

**Published:** 2025-01-09

**Authors:** Ivonne Carolina Bolaños Burgos, Júlia de Almeida Barreto, Caio Mendes Ribeiro, Pedro Henrique Oliveira Aquino, Ana Paula Bernardes Real, Aline Siqueira de Souza, Aloisio Joaquim Freitas Ribeiro, Lourdes Coral Contreras Montenegro, Gabriela Tomé Oliveira Engelmann, Erika de Oliveira Hansen, Natália Silva Dias, Andréa Teixeira Carvalho, Debora Marques de Miranda, Marco Aurélio Romano‐Silva, Luiz Armando Cunha de Marco, Bernardo M Viana, Maria Aparecida Camargos Bicalho

**Affiliations:** ^1^ Sciences Applied to Adult Health Postgraduate Program, School of Medicine, Universidade Federal de Minas Gerais (UFMG), Belo Horizonte, Minas Gerais Brazil; ^2^ Neurotec R National Institute of Science and Technology (INCT‐Neurotec R), Faculty of Medicine, Universidade Federal de Minas Gerais (UFMG), Belo Horizonte, Minas Gerais Brazil; ^3^ Cog‐Aging Research Group, Universidade Federal de Minas Gerais (UFMG), Belo Horizonte, Minas Gerais Brazil; ^4^ Undergraduate medicine, Faculty of Medicine, Universidade Federal de Minas Gerais (UFMG),, Belo Horizonte, Minas Gerais Brazil; ^5^ Neurotec R National Institute of Science and Technology (INCT‐Neurotec R), Faculty of Medicine, Universidade Federal de Minas Gerais (UFMG),, Belo Horizonte, Minas Gerais Brazil; ^6^ Cog‐Aging Research Group, 31, Minas Gerais Brazil; ^7^ Undergraduate medicine, Faculty of Medicine, Universidade Federal de Minas Gerais (UFMG), Belo Horizonte, Minas Gerais Brazil; ^8^ Neurotec R National Institute of Science and Technology (INCT‐Neurotec R), Faculty of Medicine, Universidade Federal de Minas Gerais (UFMG),, Belo Horizonte, Minas Gerais Brazil; ^9^ Cog‐Aging Research Group, Belo Horizonte, Minas Gerais Brazil; ^10^ Jenny de Andrade Faria Institute – Reference Center for the Elderly, Federal University of Minas Gerais (UFMG), Belo Horizonte, Brazil, Belo Horizonte, Minas Gerais Brazil; ^11^ Older Adult’s Psychiatry and Psychology Extension Program (PROEPSI), School of Medicine, Universidade Federal de Minas Gerais (UFMG), Belo Horizonte, Minas Gerais Brazil; ^12^ Department of Statistics, Institute of Exact Sciences, Universidade Federal de Minas Gerais (UFMG), Belo Horizonte, Minas Gerais Brazil; ^13^ Molecular Medicine Postgraduate Program, School of Medicine, Universidade Federal de Minas Gerais (UFMG), Belo Horizonte, Minas Gerais Brazil; ^14^ Jenny de Andrade Faria Institute – Outpatient Reference Center for the Elderly, Universidade Federal de Minas Gerais (UFMG), Belo Horizonte, Minas Gerais Brazil; ^15^ Older Adult Psychiatry and Psychology Extension Program (PROEPSI), Faculty of Medicine, Universidade Federal de Minas Gerais (UFMG), Belo Horizonte, Minas Gerais Brazil; ^16^ Department of Mental Health, Faculty of Medicine, Universidade Federal de Minas Gerais (UFMG), Belo Horizonte, Minas Gerais Brazil; ^17^ René Rachou Institute, Oswaldo Cruz Foundation (Fiocruz), Belo Horizonte, Minas Gerais Brazil; ^18^ Molecular Medicine Postgraduate Program, Faculty of Medicine, Universidade Federal de Minas Gerais (UFMG), Belo Horizonte, Minas Gerais Brazil; ^19^ Molecular Medicine Postgraduate Program, Faculty of Medicine, Universidade Federal de Minas Gerais (UFMG, Belo Horizonte, Minas Gerais Brazil; ^20^ Jenny de Andrade Faria Institute – Outpatient Reference Center for the Elderly, Universidade Federal de Minas Gerais (UFMG),, Belo Horizonte, Minas Gerais Brazil; ^21^ National Institute of Science and Technology Neurotec R (INCT‐MM), Belo Horizonte, Minas Gerais Brazil; ^22^ Department of Clinical Medicine, Faculty of Medicine, Universidade Federal de Minas Gerais (UFMG), Belo Horizonte, Minas Gerais Brazil

## Abstract

**Background:**

Inflammation plays a key role in Alzheimer's disease (AD) progression. Interleukin 8 (CXCL8) has emerged as an important cytokine involved in neuroinflammation. It is known as an inflammatory factor that induces a chemotactic response to sites of injury, involving the infiltration of neutrophils. In AD, interactions between CXCL8, Aβ42, and Tau suggest that CXCL8 amplifies inflammatory reactivity induced by Aβ42 and increases Tau phosphorylation and subsequent NFT formation. Our main objective was to determine the CXCL8 profile and its relationship with AD biomarkers in clinical and biological groups in a cohort of Brazilian older adults.

**Method:**

seventy‐four older adults were recruited and grouped based on different cognitive profile: Alzheimer’s dementia (n=33), Mild Cognitive Impairment (MCI) (n= 22) and normal cognition (NC) (n=19). CSF samples were collected by lumbar puncture and levels of Aβ42, p‐Tau, t‐Tau and, CXCL8 were assessed by the Luminex xMAP technique. Subsequently, based on biological criteria, the participants were classified in accordance with the AT(N) system into two groups AD continuum and non‐AD. Spearman correlation test and comparison between groups using the Mann‐Whitney U‐test and Kruskal‐Wallis's test were performed. Significant associations between CSF levels of Aβ42, p‐Tau, and p‐Tau/Aβ42 and CXCL8 were further explored in multiple linear regression analyses adjusted for age, sex, education, and APOE allele ε4 carrier status.

**Result:**

A significant correlation was found between CSF IL‐8 levels and the pTau/Aβ42 ratio (r=‐.456; p=.009) only in individuals with AD. We observed decreased CSF CXCL8 levels in the AD continuum compared to the non‐AD group [U=450.0; p=.022]. Considering clinical groups, we also observed decreased CXCL8 levels in AD group compared to the MCI group [X2 (2): 8.2; p=.016]. A significant positive association adjusted by sex, age, formal education and allele Ɛ4 was found between CXCL8 and Aβ42 (ß=.316; p=.016) and a negative association with pTau/Aß42 ratio (ß=. ‐257; p=.036).

**Conclusion:**

a decrease in CXCL8 levels was observed in the AD‐related groups and there was an increase in the MCI group, suggesting that IL‐8 may be more active in the prodromal stage of the disease and then its activity decreases as the disease progresses.